# HONORING OUR HEALTH AND HISTORY: OPERATIONALIZING CONCEPTS AND RESULTS FROM THE SHARP STUDY

**DOI:** 10.1093/geroni/igae098.1270

**Published:** 2024-12-31

**Authors:** Taryn Gordon, Launa Newby, Patrice Fuller, Charles Fennell, Raina Croff

**Affiliations:** George Fox University, Newberg, Oregon, United States; Portland State University, Portland, Oregon, United States; Oregon Health & Science University, Portland, Oregon, United States; Oregon Health & Science University, Portland, Oregon, United States; Oregon Alzheimer’s Disease Research Center, Oregon Health & Science University, Portland, Oregon, United States

## Abstract

Accessibility of mental and brain health education remains a challenge for older Black adults, hindered by cultural insensitivities within the field and discriminatory historical context. Operationalizing Black-centered theoretical concepts and narratives from the Sharing History through Active Reminiscence and Photo-imagery (SHARP) study on our website aims to leave older Black adults not only educated but empowered to address cognitive decline and engage in behaviors to mitigate it. Since 2016, 82 SHARP walkers in gentrifying areas of Portland and Seattle have generated over 400 narratives by walking 3x/week for 1, 4, or 6 months and reminiscing together, aided by image prompts about local Black history and culture. Reminiscence is audio recorded for an oral history archive. Transcribed narratives are vetted for content that may compromise participant integrity, then participant-checked. Next, narratives are coded to create a topical index for public users of the archive. SHARP researchers are now operationalizing SHARP theoretical concepts on our website and applying narratives to reach the wider Black public about brain health. The website integrates oral history from walks with tangible, culturally engaging educational content about aging, cognitive decline, and mental health. Asset-based and culturally celebratory messaging, tone, and Black history images counter the often fear- and deficit-based messaging around these topics. Framing Black health within the richness and vitality of Black history, culture, and community resilience, and through memories from other older Black adults taking agency over their health, can inspire learning and action to give readers agency in their mental and brain health.

